# Successful Treatment of Fanconi Anemia and T-Cell Acute Lymphoblastic Leukemia

**DOI:** 10.1155/2012/396395

**Published:** 2012-04-01

**Authors:** Terrie Flatt, Kathleen Neville, Karen Lewing, Jignesh Dalal

**Affiliations:** Division of Hematology/Oncology, Department of Pediatrics, Children's Mercy Hospital, 2401 Gillham Road, Kansas City, MO 64108, USA

## Abstract

Fanconi anemia is associated with an increased risk of malignancy. Patients are sensitive to the toxic effects of chemotherapy. We report the case of a patient with Fanconi anemia who developed T-cell acute lymphoblastic leukemia. He experienced chemotherapy-related complications including prolonged neutropenia, grade IV vincristine neuropathy, and disseminated aspergillosis. He was successfully treated with modified dosing of cytarabine and intrathecal methotrexate followed by allogeneic bone marrow transplant. The aspergillosis was treated with systemic antifungal treatment and surgical resection. Now 30 months after bone marrow transplant the patient is without evidence of aspergillosis or leukemia.

## 1. Introduction

 Fanconi anemia (FA) is associated with an increased risk of malignancy, and patients with FA are extremely sensitive to the toxics effects of chemotherapy [1–5]. We report the clinical case of FA with T-cell acute lymphoblastic leukemia (ALL), severe vincristine neuropathy, and disseminated aspergillosis with a successful outcome after modified chemotherapy and allogeneic bone marrow transplant.

## 2. Case Report

Our patient is a 17-year-old male who was diagnosed with FA at 13 years of age when he presented with bruising, fatigue, and pancytopenia. Physical stigmata of FA included hypopigmented skin lesions, short stature (below 3rd percentile), micrognathia, and hearing loss. Diepoxybutane (DEB) testing revealed increased chromosomal breakage. FA complementation grouping was not performed. Two years later, at the age of 15, the patient experienced bruising, epistaxis, and fatigue and was diagnosed with T-cell ALL. T-cell ALL markers included CD1+, TdT+, CD2+, CD3+, CD4+, CD5+, CD7+ and CD 8+; however, cytogenetic markers were not available. He was treated with standard 4-drug induction therapy and received vincristine, prednisone, PEG-asparaginase, and 2/4 doses of daunorubicin at an outlying hospital as per Children's Oncology Group Protocol AALL0434. His clinical course was complicated by prolonged neutropenia (ANC < 500) lasting greater than 2 months and bacterial sepsis. He developed grade IV vincristine-related neuropathy by the Balis scale grading system. Within weeks of initiating vincristine dosed at 1.5 mg/m²/dose, the patient became immobile and wheel-chair bound. He remained immobile for 12 months after vincristine therapy was terminated. He received cytarabine dosed at 125 mg/m²/dose via continuous infusion for 3 consecutive days after which the patient developed fever and profound myelosuppression. Because of new onset respiratory symptoms, a chest X-ray was obtained that revealed a right middle lobe infiltrate. CT scan of the chest showed a cavitary lesion consistent with fungal infection and he was placed on voriconazole.

**Figure 1:**
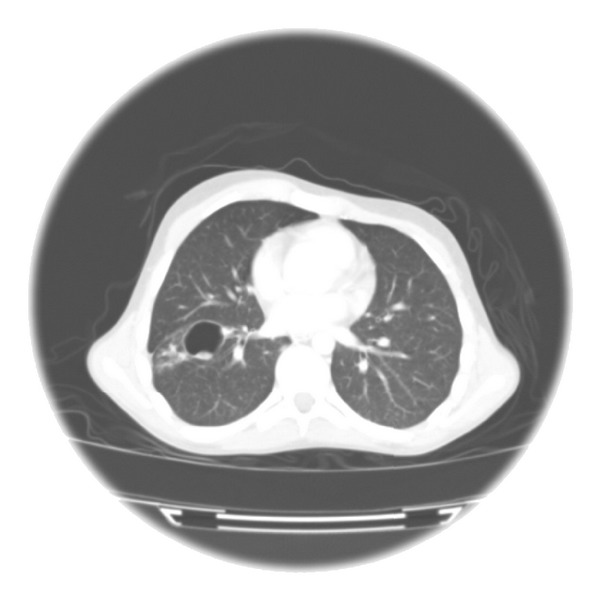
Pulmonary aspergillosis.

**Figure 2:**
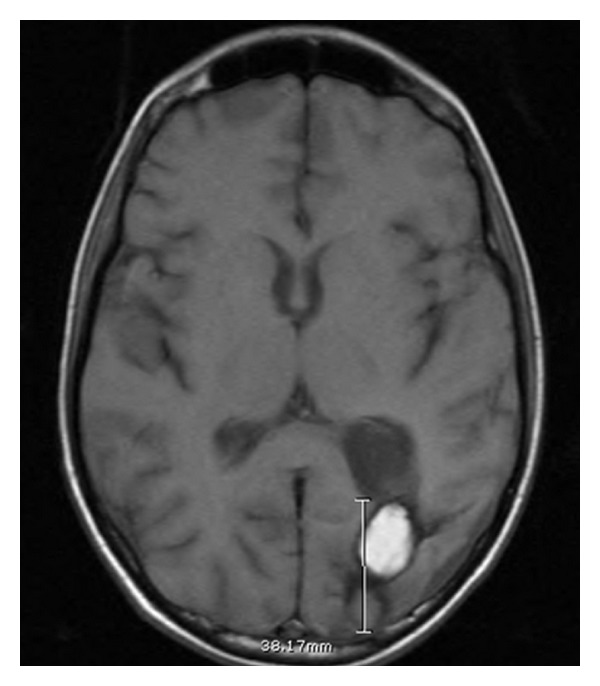
Invasive aspergillosis to brain.

 Five months after the diagnosis of T-cell ALL, the patient was referred to our center for evaluation for bone marrow transplant. Pretransplant bone marrow aspiration and cerebral spinal fluid showed no evidence of malignant cells. MRI of the brain revealed a left parietooccipital abscess and CT scan of the chest showed enlargement of the right middle lobe cavitary lesion and small bilateral nodules consistent with fungal infection (Figures 1 and 2). CT scan of abdomen and pelvis showed multiple soft tissue abscesses of the upper thighs which were drained and found to be sterile. Antifungal therapy was initiated. The patient underwent complete surgical resection of the left parietooccipital lesion and partial resection of the lung lesion. Hyphae consistent with *Aspergillus* were identified on histopathologic studies from lung and brain lesions. *Aspergillus* species was cultured from brain tissue.

 Hematologic remission was sustained with modified dosing of cytarabine and intrathecal methotrexate. Our patient received an approximately 50% dose reduction of cytarabine (60 mg/m²/dose) from the initial 125 mg/m²/dose he had received previously. Cytarabine was administered over 15 minutes for 3 consecutive days instead of a 24 hour infusion in order to reduce cell exposure to chemotherapy. Reduced dose of intrathecal methotrexate (50% reduction of age-based dosing) was given followed by leucovorin rescue. Twenty-four hours after the completion of chemotherapy, filgrastim (5 mcg/kg) was started. The patient tolerated these agents well, neutrophils recovered within 12 days, and there was no progression of aspergillosis or leukemia.

 One month after partial pulmonary aspergilloma resection and 4 months after complete parietooccipital aspergilloma resection, the patient underwent a 10/10 matched unrelated donor bone marrow transplant using a modified reduced-intensity preparative regimen. The patient received voriconazole 200 mg orally daily and micafungin 100 mg IV daily for 4 months prior to BMT. Galactomanman levels remained negative, and radiographic studies of the brain and chest showed improvement and no new fungal foci. The BMT preparative regimen included fludarabine 140 mg/m^2^, cyclophosphamide 40 mg/kg, thymoglobulin 6 mg/kg (total doses), and 450 cGy TBI. 3 × 10^6^ CD34+ stems cells were obtained from the donor's bone marrow. T-cell depletion was achieved using rabbit ATG. GVHD prophylaxis included tacrolimus and mycophenolate. Neutrophils engrafted by day 14. There were no acute transplant-related complications. He received voriconazole for 6 months after BMT. At the time of this writing, 30 months after BMT the patient is without evidence of recurrent aspergillosis or leukemia, and continues to do well.

## 3. Discussion

 Hematological malignancies and solid tumors associated with Fanconi anemia are well described in the literature [1]. Lymphoid leukemia is rare as compared to acute myeloid leukemia (AML) in patients with FA. In Alter's review from 1927 to 2001, only 7 cases of ALL were reported as compared to 109 cases of AML [1]. Kutler et al. reported 755 persons from North America with confirmed FA. From this population, 178 malignant neoplasms were identified and 60 cases were AML while only 5 were ALL [2]. We identified two case reports of FA and T-cell ALL. Both patients successfully achieved remission but their long-term outcome is unknown [3, 4]. One case of T-cell lymphoblastic lymphoma was reported, and the patient experienced severe myelosuppression and died of progressive disease [5]. All patients in these case reports experienced profound myelosuppression, but severe vincristine toxicity was not reported.

 One of the principal clinical challenges faced while treating this patient was maintaining leukemic remission while providing treatment for disseminated aspergillosis and searching for bone marrow transplant donor. There is a dearth of literature regarding modified chemotherapy dosing in FA patients, especially in the face of disseminated aspergillosis. It is well known that FA cells are sensitive to the effects of reactive oxygen species that are created by both alkylating agents and anthracyclines [6]. Many of the common DNA-damaging agents (cyclophosphamide and doxorubicin) used to treat ALL can be toxic in this patient population as demonstrated by clinical case reports and in vitro cell line studies. While patients with FA are hypersensitive to these agents, Yule et al. also demonstrated that the pharmacokinetics of cyclophosphamide, for example, is different between control groups and the FA group [7]. The median clearance of cyclophosphamide was much lower in the FA group compared to the control group (0.6 L/m²/hr versus 3.2 L/m²/hr), and the *t*(1/2) was increased in the FA group compared to the control group (8.1 hrs versus 2.4 hrs) indicating that FA patients may experience more drug toxicity because of differences in their ability to metabolize and eliminate the drug and consequently experience increased exposure time to the drug [7].

 Mehta et al. describe the use of dose-adjusted cytarabine and fludarabine with limited toxicity in the treatment of AML in patients with underlying FA prior to BMT [8]. Cytarabine has also shown activity against resistant lymphoblastic leukemias. In vitro studies comparing the effects of fludarabine, cytarabine, and cyclophosphamide on chromosomal breakage in FA cells revealed that cytarabine and fludarabine did not correlate with increased chromosomal breaks in FA cells when compared to normal host cells inferring that these may be safer treatment options in this patient population [9]. Cytarabine was the chemotherapeutic agent that we chose, and he tolerated modified dosing of this agent well.

 This patient experienced severe grade IV vincristine peripheral neurotoxicity. Three months after BMT, the patient showed significant improvement in his neuropathy and began to ambulate without assistance. While vincristine-related neurotoxicity is well known, the drug's toxicity profile has not been established in patients with FA. Rosselli et al. have described low levels of IL-6 and increased levels of tumor necrosis factor-alpha (TNF-*α*) in cultured lymphocytes of patients with FA [10]. Briot has suggested that the increase in TNF-*α* secretion in FA cell lines may be related to defects in the stress response signaling pathways and may increase the production of reactive oxidative species and thus induce cytotoxic properties including DNA damage and cell death [11]. The hypersensitivity to mitomycin C that was observed in FA cells was partially corrected with the addition of IL-6 and TNF-*α* antagonists to FA lymphocytes [11]. Recently, IL-6 has been shown to be a protective agent and a nerve growth factor stimulant in the murine model for chemotherapy-induced neuropathy with vincristine, paclitaxel, and cisplatin [12]. While the vinca alkaloids are not a drug class that has specifically been associated with severe toxicity in FA patients, this case suggests that dose adjustments of vincristine may need to be considered in this patient population. In addition, drugs that have the potential to increase the toxic effects of vincristine or interfere with its metabolism may need to be used with special caution in FA patients who are receiving vincristine.

 Another unique clinical challenge presented by our patient is that he presented with disseminated *Aspergillus* infection in the lungs and CNS. Mortality associated with invasive fungal infections, especially in the CNS, in the immunocompromised host has ranged from 60% to almost 100% [13–15]. In the past, some institutions would have considered this complication alone as a contraindication for bone marrow transplantation [15]. Over the last decade, systemic treatments have improved dramatically, and studies have shown that voriconazole penetrates the CNS and has been efficacious in the treatment of invasive fungal disease [16, 17]. Surgical resection of aspergillomas from both the CNS and lungs has been reported as an effective strategy to eradicate fungal disease [17–19]. This case demonstrates that surgical resection along with systemic treatment of fungal disease for greater than 30 days may play a pivotal role in treating patients before proceeding to BMT, and that such patients can experience a successful outcome [20].

 This unique case describes the treatment of a patient with FA, T-cell ALL, and multiple medical issues including invasive aspergillosis. Collectively, the medical strategies that we utilized resulted in the successful treatment of a highly complex patient. This case suggests that modified chemotherapy dosing with non-DNA cross-linking agents may sustain bone marrow remission and may decrease chemotherapy-related toxicities. For FA patients with an underlying malignancy who may not be able to proceed immediately to BMT, future studies will need to focus on the pharmacokinetics and genetic polymorphisms that can influence drug metabolism in FA patients in order to provide optimal chemotherapeutic choices and to develop better chemotherapeutic-dosing guidelines. Given the difference in levels of IL-6 and TNF-*α* in patients with FA, testing of IL-6 and TNF-*α* levels both before and after the transplant in FA patients could be useful in order to better elucidate the clinical role of these cytokines in this patient population. Because patients with FA are rare, single or case series that focus on the treatment of malignancies in this patient population will need to be encouraged until clinical trials are developed to address these clinical conundrums.
